# Genomic Insights into Dementia: Precision Medicine and the Impact of Gene-Environment Interaction

**DOI:** 10.14336/AD.2024.0322

**Published:** 2024-10-01

**Authors:** Anjali Tripathi, Vinay Kumar Pandey, Garima Sharma, Ashish Ranjan Sharma, Anam Taufeeq, Abhimanyu Kumar Jha, Jin-Chul Kim

**Affiliations:** ^1^Department of Biotechnology, Sharda School of Engineering and Technology, Sharda University, Greater Noida, Uttar Pradesh, India.; ^2^Division of Research & Innovation (DRI), School of Applied & Life Sciences, Uttaranchal University, Dehradun, Uttarakhand, India.; ^3^Department of Biomedical Science & Institute of Bioscience and Biotechnology, Kangwon National University, Chuncheon 24341, Republic of Korea.; ^4^Institute for Skeletal Aging & Orthopedic Surgery, Hallym University-Chuncheon Sacred Heart Hospital, Chuncheon-si, 24252, Gangwon-do, Republic of Korea.; ^5^Department of Biotechnology, Faculty of Engineering and Technology, Rama University, Kanpur, Uttar Pradesh, India

**Keywords:** Dementia, Precision Medicine, Gene-environment interactions, Neurodegenerative Disorders, Genomic Insight

## Abstract

The diagnosis, treatment, and management of dementia provide significant challenges due to its chronic cognitive impairment. The complexity of this condition is further highlighted by the impact of gene-environment interactions. A recent strategy combines advanced genomics and precision medicine methods to explore the complex genetic foundations of dementia. Utilizing the most recent research in the field of neurogenetics, the importance of precise genetic data in explaining the variation seen in dementia patients can be investigated. Gene-environment interactions are important because they influence genetic susceptibilities and aid in the development and progression of dementia. Modified to each patient's genetic profile, precision medicine has the potential to detect groups at risk and make previously unheard-of predictions about the course of diseases. Precision medicine techniques have the potential to completely transform treatment and diagnosis methods. Targeted medications that target genetic abnormalities will probably appear, providing the possibility for more efficient and customized medical interventions. Investigating the relationship between genes and the environment may lead to preventive measures that would enable people to change their surroundings and minimize the risk of dementia, leading to the improved lifestyle of affected people. This paper provides a comprehensive overview of the genomic insights into dementia, emphasizing the pivotal role of precision medicine, and gene-environment interactions.

## 1. Introduction

The neurological condition known as dementia is intricate and multidimensional, posing serious difficulties for individuals as well as medical personnel. Precision medicine appears to be a viable approach to comprehending and even treating dementia as we learn more about its genetic makeup [1]. Numerous genes that control different facets of brain development and function are encoded in the human genome. Recent developments in genomic research have illuminated the complex interactions between genes and the environment, offering important new insights into the hereditary basis of dementia. Finding the genetic risk factors linked to the onset of dementia is one of the main goals of genomic research in this field. Several studies have linked genes to the aetiology of different types of dementia, such as vascular dementia (VaD) and Alzheimer's disease (AD) [2-3]. The discovery of these genetic markers expands our knowledge of the underlying mechanisms and creates new avenues for focused treatment interventions [2]. In the context of dementia, precision medicine a paradigm that customizes medical care to each patient's unique characteristics holds considerable potential [4, 5].

High-throughput sequencing technology has completely changed the science of genomics by allowing scientists to look at the entire genome and find minute differences that could be linked to a person's susceptibility to a disease [6]. Common genetic variations linked to a higher risk of dementia have been found in large part to genome-wide association studies (GWAS) [4]. These variations, which are frequently found in or close to genes related to immunological response, inflammation, and neuronal function, offer important hints concerning the molecular pathways connected to dementia. The existence of particular risk genes does not entirely define the genetic landscape of dementia. Relationships between genes and environment are important in determining a person's risk of dementia [7, 8]. Genetic risk factors can have an effect that is modulated by environmental factors, including nutrition, lifestyle, and exposure to pollutants. For creating individualized interventions that consider both genetic and environmental factors, it is imperative to comprehend these connections. For instance, lifestyle changes that have been demonstrated to reduce the risk of cognitive decline, such as regular exercise, and a nutritious diet, may be beneficial for someone with a genetic predisposition to dementia [9,10]. Genetic testing's ability to forecast a person's likelihood of acquiring dementia raises concerns regarding permission, privacy, and the psychological effects of such data. It is critical to strike a balance between expanding scientific understanding and ensuring people's safety [11,12,13].

The intricate relationship between genetics and environment, in addition to the moral dilemmas associated with genetic data, emphasizes the necessity of an integrated and interdisciplinary strategy to address dementia's problems [10,14,15]. The review paper's goal is to comprehensively examine the genetic foundations of dementia, with a particular emphasis on precision medicine and the complex interactions between genes and environments. It seeks to offer a broad insight into upcoming studies and treatment modalities.

## 2. Significance of Genetics in Dementia

To recognize the nuanced causes of neurodegenerative diseases, researchers have been negotiating the extensive and complex genetic topography of dementia. The risk, enhancement, and course of various neurodegenerative illnesses are greatly influenced by the complex and varied terrain of dementia’s genetics [14]. The complex web of various disorders is largely shaped by the hereditary foundations of dementia. Notably, a few genes have come to light as important participants in the genetic architecture of dementia, including the presenilins (PSEN1 and PSEN2), the apolipoprotein E (APOE) gene, and microtubule-associated protein tau (MAPT) [16]. One significant genetic risk factor for the late onset of AD is APOE. Autosomal dominant inheritance patterns are frequently seen in familial cases of AD, highlighting the impact of genetic abnormalities in the spreading of the disease. Except for AD, dementia is a genetic field that encompasses a range of neurodegenerative conditions, such as Lewy body dementia (LBD), frontotemporal dementia (FTD), and VaD. Distinct genetic signatures are displayed by each of these types, which impact the clinical manifestation, course, and reaction to therapy [1]. Although many genetic factors may be common in all forms of dementia, others are exclusive to subtypes, illustrating the diversity within the more general category of neurodegenerative illnesses. Numerous common genetic variants linked to dementia have been found by GWAS, indicating a polygenic nature in which multiple genetic factors jointly contribute to illness risk. In this section of the article, we will be discussing the genetic basis of dementia.

### 2.1. Genetic variants associated with different forms of dementia

The number of cases of dementia is rapidly increasing across the globe, although very little information is known about the precise origins of the various forms of dementia. It is believed that they are inherited as well as environmental. AD, VaD, and FTD are the three most prevalent forms of dementia, each with its distinct genetic foundation [11]. In 10 million new instances of dementia, approximately two-third are identified each year is caused by AD. One known genetic risk factor for AD is the APOE gene, which is found on chromosome 19. The APOE ε2 allele may protect the condition, but people with the APOE ε4 allele are more likely to get it. Other genes have also been linked to AD in addition to APOE. Early-onset familial AD, a rare variant of the disorder that usually appears before the age of 65, is linked to PSEN1 and PSEN2. These genes' mutations affect the method by which the amyloid precursor protein (APP) is processed, which results in the buildup of beta-amyloid plaques in the brain, a characteristic of AD. Missense mutations, tiny insertions, deletions, and genomic deletions, particularly in PSEN1, are among the PSEN gene spectrum's features [16]. The severe types of AD with 100% penetrance are caused by PSEN1 mutations, and the disease can start as early as age 25. The missense mutations result in modified proteins that obstruct the γ-secretase complex's fusion. This, in turn, changes the way gene APP is processed, which raises the Aβ42/Aβ40 ratio [17]. The 17 exons that make up the APP gene, which codes for this protein, result in several isoforms due to alternative splicing of exons 7 and 8 [14]. In these, three isoforms 695, 751, and 770 are specifically related to AD and are expressed only in the central nervous system. The final two exons, 16 and 17, code for the part of APP that becomes the Aβ fragment following proteolytic processing [18]. Remarkably, 73 variants (of which 32 are harmful) have been found, in which the Val717Ile/London mutation is the most common [19, 14]. Increases in total and phosphorylated Tau levels in neurons, as well as the Aβ42/Aβ40 ratio, are caused by mutations in the APP gene [20, 14]. While not all trisomy, 21 people go on to acquire AD, the majority do show early pathology alterations like those of the illness [21]. This implies that the APP genetic load increase, which has previously been noted in familial AD patients, may not always be the source of the disease's onset [14].

APOE is the primary gene consistently linked to sporadic AD, increasing the risk of AD three- to eight-fold [22]. Among healthy populations, 50 to 90 percent have ApoE-ε3 (Cis112Arg158), the physiological isoform. The primary variations in the APOE gene are caused by alleles that translate different protein isoforms that are only differentiated by a single amino acid substitution at positions 112 and 158: The ApoE-ε2 isoform (Cis112Cis158) has been linked to the manifestation of hyperlipoproteinemia type III [14]. On the other hand, the ApoE-ε4 isoform (Arg112Arg158) has been linked to atherosclerosis [23,24], and 40-65% of AD patients have at least one copy of this allele. It should be noted that not everyone carrying copies of this gene becomes ill; it merely serves as a susceptibility risk factor [14,21]. Competitively binding to Aβ receptors on the surface of astrocytes, such as the low-density lipoprotein receptor-related protein 1; LRP1, APOE prevents Aβ absorption, influences its clearance, and stimulates the first seeding of fibrillar Aβ deposition [20, 25]. There is mounting evidence that APOE affects microglial responses to AD-related diseases as well as tau-mediated neurodegeneration [23]. A study found that the APOE3ch variation might play a locally specific role in altering the impact of Tau and, consequently, the degree, course, and clinical manifestation of AD [24]. This indicates that APOE polymorphisms might have a significant predisposing effect on AD and impact the processing of Tau protein [14].

#### 2.1.1 Vascular Dementia (VaD)

VaD, the second most frequent kind of dementia when compared to other types of dementia including AD, Parkinson's disease, and frontotemporal lobar degeneration, its hereditary component is still poorly known [26]. The VaD, caused by reduced blood supply to the brain, is impacted by a complicated interaction between environmental and hereditary variables. VaD has been linked to several genes, and it is vital to consider the possibility that these genes will be passed down to future generations [27]. The heritability of VaD has only been studied in one study including 24 twins till 2017, and that study was unable to detect a substantial genetic component. Given the small sample size and the variation in VaD definitions, a cautious interpretation is necessary, even though this would imply that the environment has a greater influence. Moreover, several lines of evidence suggest that there may be a significant hereditary component to VaD [28].

The APOE gene, specifically its ε4 allele, is one of the prominent genes linked to VaD. The ε4 variant of APOE is known to be a substantial genetic risk factor for several cardiovascular and neurological illnesses, including VaD and AD [29]. APOE is essential for lipid metabolism. People who have the APOE ε4 allele are more likely to develop cerebral small artery disease and vascular lesions over time, which can lead to cognitive deterioration [22, 30]. VaD has been linked to variations in blood vessel form and function that are caused by genetics. Nitric oxide synthase (NOS3) and endothelin-1 (EDN1) are two examples of genes with polymorphisms that may affect vascular health and have a role in the pathophysiology of VaD [31]. Vascular dysfunction may result from changes in the activity of these genes, which are essential for controlling endothelium homeostasis and blood vessel tone [31]. Renin-angiotensin system, a major blood pressure regulator, involves genes like ACE (angiotensin-converting enzyme) and AGT (angiotensinogen). Variants in these genes may raise a person's risk of VaD by making them more susceptible to hypertension [32]. The most common heritable form of VaD is cerebral autosomal dominant arteriopathy with subcortical infarcts and leukoencephalopathy (CADASIL). Frequent symptoms include aura-accompanied migraine, repeated strokes, and mental health issues. It is caused by NOTCH3 gene (19q12) mutations. While deletions, duplications, splice site mutations, and deletion/insertions have all been documented, missense mutations (exon 2-24) account for the vast majority of the about 230 harmful mutations that have been reported thus far. Vascular smooth muscle cells' ability to function is impacted by all these mutations, which are in the tandem EGF-like repeat region of the Notch3 receptor and result in either a gain or deletion of a cysteine residue [33,34]. It is still up for debate whether mutations that cause loss of function, such as nonsense mutations or frame-shift deletions, and non-cysteine variations, can cause CADASIL [35]. Remarkably, in GWAS involving 466 European CADASIL patients, the burden of white matter hyperintensities (WMHs) in CADASIL has been linked to many genetic variations with negligible effect, rather than being associated with the various NOTCH3 mutations [34]. A single Italian study that found a significant correlation between the rs1333049 polymorphism in 9p21.3 and VaD [34] has served as the basis for linkage analyses in VaD. However, most of the genetic research in Sporadic VaD has focused on association studies with candidate genes involved in stroke and AD and/or in pathophysiological mechanisms critical to VaD [26]. Most of these studies have so far shown slightly significant associations with VaD (0.01 < p < 0.05), and very few have been replicated [36,37,38]. Complicating our understanding of familial propensity to VaD is the possibility that these genetic factors will be passed on to the next generation. Although VaD in offspring is not always the result of genetic inheritance, it can be increased by the existence of specific risk genes [38,39]. To enable early treatments and preventive measures, genetic counseling, and risk assessment based on a person's genetic profile may become crucial elements of customized healthcare.

#### 2.1.2 Frontotemporal dementia (FTD) and Lewy body dementia (LBD)

FTD and LBD are two different neurodegenerative diseases, each with its own specific clinical and pathological characteristics. Numerous studies have been conducted on the heritability of various disorders, revealing specific genes that are involved in their development [40]. Numerous genetic variables have been linked to LBD, with particular attention paid to the SNCA gene. Only three genes APOE, GBA, and SNCA have been conclusively shown to be implicated in LBD thus far. LDB traits can be caused by or modulated by variation in the SNCA gene. It is also recognized that the LBD risk variables that have been established also carry the risk for AD (APOE) or Parkinson's Disease (PD) (GBA, SNCA) [41]. Alpha-synuclein, a protein that forms aberrant aggregates known as Lewy bodies, which are a distinguishing feature of LBD, is encoded by this gene. The SNCA gene's importance in the pathophysiology of the disease has been highlighted by the links it has made to both familial and sporadic forms of LBD [42]. There are concerns regarding familial susceptibility because of the possibility that these genetic variants will be passed down to the following generation. There are no genetic risk factors in common between AD and PD [41]. This is consistent with a previous study that found no indication of a correlation between PD and AD but did find a genetic correlation of 0.578 (SE ± 0.075) between DLB and AD and 0.362 (SE ± 0.107) between DLB and PD. AD and DLB have a strong correlation with the APOE locus. The association between AD and DLB decreased to 0.332 once this locus was eliminated from the study, which is not substantially different from the connection between LDB and PD [41, 42]. A genetic component of FTD, which is defined by gradual impairment to the brain's frontal and temporal lobes. It is well known that several genes, including MAPT, GRN, and C9orf72, are involved. One of the factors linked to the tauopathy subtype of front temporal dementia is mutations in the MAPT gene, which codes for tau protein [43]. Similarly, family types of FTD have been associated with mutations in the C9orf72 gene and the GRN gene, which generates progranulin. The build-up of aberrant proteins in the brain caused by these hereditary variables contributes to neurodegeneration and cognitive impairment. In the context of LBD and FTD, the possibility that these genetic variables be passed down to the next generation emphasizes the importance of genetic counseling and risk assessment [44]. Table 1. explains several genes involved in AD, FTD, and VaD including their physiological and pathological role for precise understanding.

Although certain mutations might greatly increase the risk for family members, not all cases of these dementias are directly inherited. Being aware of the carriers of certain genetic variations enables proactive management, such as early detection and treatment [1]. Researchers are still untangling the complex interplay between genetic and environmental factors and how they influence the development and progression of different diseases. With age-related disorders such as dementia, scientists are also investigating the role of genetic factors in early illness onset and progression. The aim is to establish any underlying processes and potential therapeutic targets that guide how the disease develops [45]. By looking at the DNA sequences that are different in people, scientists have used cutting-edge genomic technology to investigate the genetic makeup of dementia patients and find genetic factors that are responsible for developing and being susceptible to getting the disease. These outside factors involve their environment -- lifestyle choices, exposure to chemicals, socioeconomic status, and where they are located geographically. Extensive datasets and studies that look over the long term have unveiled how intricate gene-environment relationships can shape how dementia and other age-related illnesses progress [46]. By sussing out these complex interactions, which are dependent on the genetic make-up and extent and nature of environmental exposure on an individual basis, scientists hope to develop ways in which illnesses can be prevented, diagnosed and treated that depend on the individual's specific biology and environment [47].

**Table 1:** Physiological and pathological role of Various genes involved in numerous types of Dementia.

Associated Dementia	Gene Name	Physiological Role	Pathological Role	Reference
AD	APOE	Lipid transport and metabolism	Increased risk of beta-amyloid accumulation in AD	[45]
AD	PSEN1 and PSEN2	Presenilins involved in APP processing	Early-onset familial AD mutations	
FTD	MAPT	Microtubule-associated protein tau	Tauopathy in FTD and AD	[46]
FTD	GRN	Progranulin, involved in inflammation and neuronal survival	Mutations linked to familial FTD	[47]
FTD	C9orf72	Chromosome 9 open reading frame 72	FTD and amyotrophic lateral sclerosis	
AD	CLU	Cluster in, involved in cell aggregation and apoptosis	Genetic variant associated with increased Alzheimer's risk	[48]
AD	BIN1	Bridging integrator 1, regulates endocytosis	Implicated in tau pathology and AD progression	[49]
AD	CR1	Complement receptor 1, part of the immune system	Genetic variants associated with increased Alzheimer's risk	[50]
AD	CD33	Sialic acid-binding immunoglobulin-like lectins	Modulates microglial function and affects beta-amyloid clearance	[51]
AD	MS4A	Membrane-spanning 4-domains, subfamily A	Involved in immune response and associated with Alzheimer's risk	
AD	SORL1	Sortilin-related receptor 1, regulates APP trafficking	Implicated in late-onset AD	
AD	TREM2	Triggering receptor expressed on myeloid cells 2	Associated with microglial function and Alzheimer's risk	[52]
AD	INPP5D	Inositol polyphosphate-5-phosphatase D	Involved in immune response and linked to Alzheimer's susceptibility	
AD	ABCA7	ATP-binding cassette transporter A7	Implicated in lipid metabolism and associated with Alzheimer's risk	[53]
AD	EPHA1	Ephrin type-A receptor 1, regulates cell signaling	Genetic variant associated with increased Alzheimer's risk	[54]
AD	CD2AP	CD2-associated protein, involved in cell adhesion	Linked to synaptic dysfunction and AD progression	
AD	PICALM	Phosphatidylinositol-binding clathrin assembly protein	Involved in endocytosis and implicated in Alzheimer's risk	[55]
AD	SORCS1	Sortilin-related VPS10 domain containing receptor 1	Associated with AD susceptibility	[56]
VaD	NOTCH3	Notch receptor 3, involved in cell signaling	Mutations linked to CADASIL	[57]
VaD	HTRA1	High-temperature requirement A serine peptidase 1	Associated with small vessel disease and increased risk of VaD	[58,59]
VaD	ACE	Angiotensin-converting enzyme	Involved in blood pressure regulation, genetic variants associated with VaD risk	[60]
VaD	MTHFR	Methylenetetrahydrofolate reductase	Role in homocysteine metabolism, genetic variants associated with increased risk of VaD	

Clusterin (CLU), Apolipoprotein E (APOE), presenilin 1 and 2 (PSEN1 and PSEN2), Bridging Integrator 1 (BIN1), complement component receptor 1 gene (CR1), sialic acid binding Ig-like lectin 3 Siglec-3 or (CD33), Membrane-spanning 4-domains subfamily A (*MS4A*), Neuronal sortilin-related receptor (SORL1), Triggering receptor expressed on myeloid cells 2 (TREM2), Inositol polyphosphate-5-phosphatase (INPP5D), Adenosine triphosphate-binding cassette transporter subfamily A member 7 (ABCA7), Erythropoietin-producing hepatocellular (Eph) family (EPHA1), CD2 associated protein (CD2AP), Phosphatidylinositol binding clathrin-assembly protein (PICALM), Sortilin Related VPS10 Domain Containing Receptor 1 (SORCS1), Notch Receptor 3 (NOTCH3), HtrA Serine Peptidase 1 (HTRA1), Angiotensin I converting enzyme (ACE), Methylenetetrahydrofolate reductase (MTHFR), Microtubule Associated Protein Tau (MAPT), Granulin Precursor (GRN), chromosome 9 open reading frame 72 (C9orf72)

### 2.2. Genetic Factors of Dementia and Their Functional Implications

The aetiology of dementia is mostly influenced by genetic variables, which also affect how different neurodegenerative illnesses proceed and are susceptible to developing at different ages. The APOE gene is one of the many factors linked to AD, the most prevalent type of dementia, and it is important. There are three primary alleles of the APOE gene: ε2, ε3, and ε4. While the ε2 allele seems to have some protective effects, those who carry the ε4 allele are more likely to develop AD [61]. These allelic differences have functional implications because they affect lipoprotein metabolism and amyloid-beta (Aβ) peptide clearance, two important pathways in AD [62].

An increased risk of Aβ accumulation in the brain, a characteristic feature of AD, has been linked to the APOE ε4 allele. Research has indicated that carriers of APOE ε4 typically display elevated Aβ accumulation and decreased clearance, which fosters the development of neurotoxic Aβ plaques. Affected neural structure and function have been connected to the APOE ε4 variation, which may explain the synaptic malfunction and cognitive decline seen in Alzheimer's patients [63]. Comprehending the functional consequences of genetic variations in APOE illuminates possible avenues for therapeutic intervention, to regulate Aβ metabolism and alleviate neurodegenerative mechanisms. Other genetic variables contribute to the complex landscape of dementia in addition to APOE. PSEN and PSEN gene mutations have been linked to early-onset AD in family forms [64]. These genes are essential for the processing of the APP, which affects the generation of Aβ. The influence of PSEN1 and PSEN2 mutations on Aβ42, the more lethal form of Aβ, has been emphasized by functional studies, highlighting the genetic basis of Aβ-related neurodegeneration. Understanding the functional effects of these mutations is crucial for figuring out the mechanisms underlying AD with an early onset and developing viable treatment strategies [61]. Apart from the pathways linked to Aβ, hereditary variables also play a role in the pathophysiology of dementia, including tau pathology and neuroinflammation. FTD is one of the tauopathies linked to the MAPT gene, which encodes tau protein. Neurofibrillary tangles are the result of aberrant tau protein aggregation, which is the cause of tau disease [65]. Variants in the MAPT gene affect the isoform composition of tau and play a role in the pathological aggregation of tau. Comprehending the functional implications of MAPT variations opens opportunities for tailored treatment therapies and sheds light on the molecular mechanisms driving tauopathies. The modulation of neuroinflammatory responses in AD has drawn attention to the TREM2 gene, which encodes for triggering receptors expressed on myeloid cells [66]. TREM2 is involved in the phagocytosis of Aβ and cellular debris and is expressed in microglia, the immune cells that make up the central nervous system. Variants in TREM2 affect microglial activity and the neuroinflammatory milieu in the brain; they have also been linked to an increased risk of AD [62]. The functional consequences of TREM2 variations highlight the complex interaction between neuroinflammation and genetic variables in the aetiology of dementia. The APOE, PSEN1, PSEN2, MAPT, and TREM2 genes are among the genes that demonstrate the functional consequences of genetic variations [67]. These insights are crucial for understanding the molecular mechanisms underlying the pathophysiology of dementia. The potential for precision medicine techniques customized to individual genetic profiles is becoming more and more intriguing as genomic research progresses, revealing the complexities of genetic variables in dementia [64].

## 3. Implication of Precision Medicine in Dementia

Precision medicine plays a critical role in transforming the care of people with dementia. By customizing treatment plans to each patient's unique genetic, biochemical, and lifestyle characteristics, Comprehending the complex differences in genetic composition allows for tailored treatments, maximizing efficiency and reducing adverse effects [1]. This individualized approach is extremely important when it comes to dementia, as there are many different causes of cognitive loss [1]. Precision medicine identifies genetic markers linked to various types of dementia, facilitating early diagnosis and treatments. These focused therapies seek to improve therapeutic results, impede the advancement of the disease, and eventually raise the standard of living for dementia patients. The field of precision medicine is developing, which gives hope for more individualized and efficient dementia care techniques. In this section of the article, we will discuss the significance of precision medicine in depth.

### 3.1. Application of Precision Medicine Approaches to Dementia

**Figure 1.**
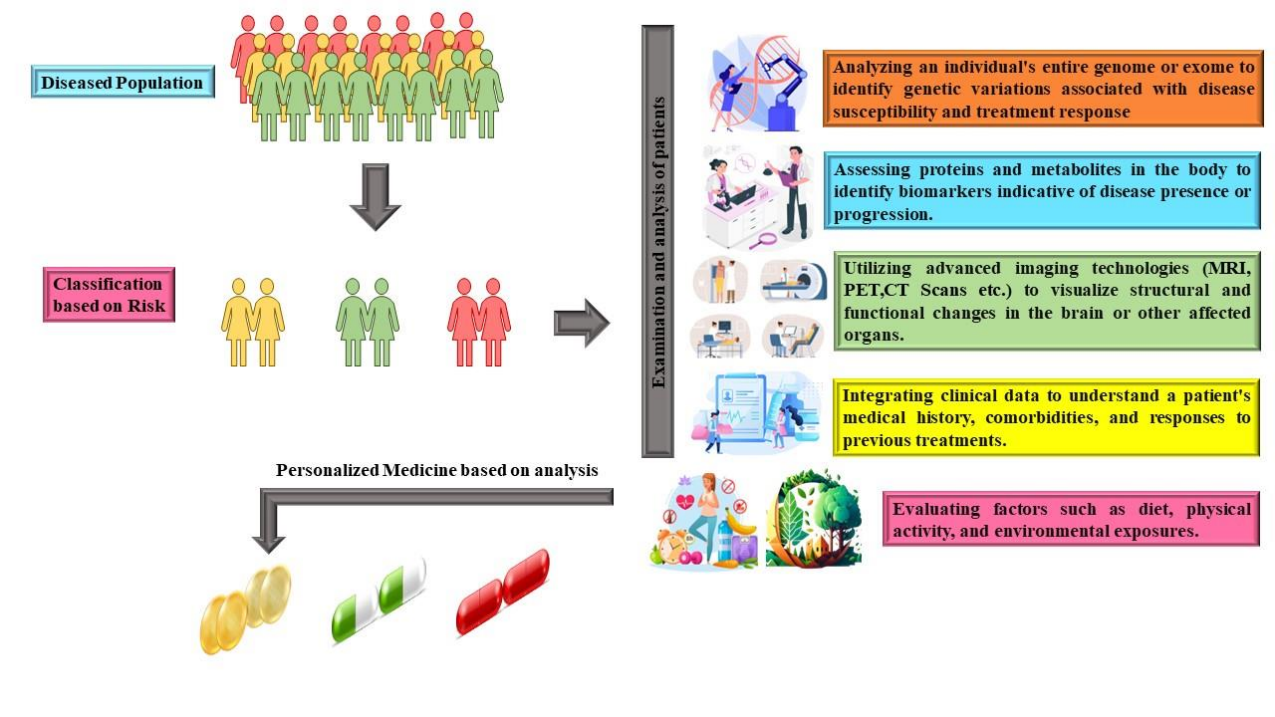
Precision Medicine for prevention of disease. In the case of Dementia, it uses biomarkers, neuroimaging techniques, and genome analysis to identify and categorize individuals based on specific illnesses. This makes it possible to create targeted medicines more personalized.

Precision medicine, which is often referred to as personalized medicine, is an innovative approach to healthcare and medical treatment that considers the distinctive genetic, environmental, and lifestyle variations of each patient [67]. Precision medicine is based on the core principle of customizing medical interventions and care to the individual needs of each patient, rather than using a one-size-fits-all approach. Customizing medical care according to a patient's genetic composition, lifestyle, and surroundings as shown in Figure 1. is known as precision medicine [68]. It acknowledges that every patient is different and that a treatment plan that benefits one may not benefit another or may even be harmful. This strategy seeks to minimize negative reactions and side effects while maximizing the efficacy of treatments. The examination of a person's genetic data is the basis of precision medicine. Finding genetic variants that may affect a disease's propensity to develop, course, and responsiveness to therapy is one aspect of this. Next-generation sequencing and other genomic technology advancements have made a substantial contribution to our understanding of individual genetic patterns [69]. Clinicians are better equipped to recommend treatments that have a higher chance of success when they are aware of the precise molecular processes involved. The influence of lifestyle and environmental factors on health is acknowledged by precision medicine. It considers outside factors including nutrition, physical activity, and exposure to pollutants to give a thorough picture of a person's health profile [68].

The paradigm of precision medicine, which customizes medical care to each patient's unique needs, is revolutionizing the study and treatment of dementia. Because dementia has a wide range of underlying causes and symptoms, it presents challenges. A thorough understanding of the genetic, molecular, and environmental components of dementia is necessary to apply precision medicine techniques to the condition [70]. This individualized approach has the potential to enhance outcomes for dementia patients by enabling more precise diagnosis and focused treatment. A comprehensive investigation of the patient's genetic composition is the first step in precision therapy for dementia. According to genomic studies, certain genetic variants are linked to a higher chance of developing dementia, including FTD, VaD, and AD. In at-risk older individuals, it is intended to detect the entire spectrum of specific biochemical abnormalities well in advance of the onset of the initial clinical symptoms [71]. The application of Precision medicine in the domains of neuroscience, psychiatry, and neurology is expected to induce a fundamental change in the strategy for managing brain disorders, with an increased emphasis on timely identification and efficacious early interventions. With a particular emphasis on individualized care, prevention techniques can be implemented prior to the occurrence of significant disease progression [72]. Precision medicine’s primary aim is to establish novel frameworks that facilitate the identification, categorization, prognosis, treatment, and prevention of neurodegenerative disorders through the utilization of distinct physiological attributes that are reflected in multidimensional potential biomarkers. Investigations in the fields of neurogenetics and neuroepigenetics have yielded evolving insights into biomarkers for AD over the past two decades [1]. Additionally, neurophysiology and structural, functional, and metabolic imaging, as well as neurochemistry—which has been applied to cerebrospinal fluid (CSF) and blood —have contributed to this understanding [73,1].

Genetic testing can yield important insights into a person's predisposition to types of dementia, facilitating early detection and customized risk mitigation [74]. The goal of precision medicine is to locate and utilize biomarkers, which are molecular markers of biological processes connected to dementia, as stated above. These could consist of metabolites, proteins, or other things that can be measured within the body. Biomarkers for the onset or progression of dementia can be found with the help of advanced imaging techniques and the study of CSF. These biomarkers allow for earlier and more precise diagnosis, which expedites therapy planning and intervention. There are many different aetiologies for dementia, making it a complex condition with variable symptom presentation and progression [75]. Biomarker-based categorization of therapeutic intervention is critical to precision medicine, with prototype medications such as imatinib and trastuzumab serving as the foundation after demonstrating exceptional efficacy in specific patients. The biomarkers T-Tau, p-Tau, and Aβ-42 have exhibited the highest degree of reliability and should be utilized in both clinical practice and research. The accuracy of these biomarkers ranges from 85% to 90% [1]. Dementia can now be classified into subtypes according to different genetic and molecular profiles because of precision medicine. Gaining an understanding of these subtypes is essential for creating focused treatments that are matched to the underlying causes and may improve the effectiveness of treatment. The variability of dementia demands that we move away from one-size-fits-all approaches to treatment [76]. It is possible to tailor targeted drugs, lifestyle changes, and cognitive therapy to each patient's unique processes of cognitive decline [77]. Clinicians can forecast a patient's risk of dementia by using genetic and biomarker data acquired using precision medicine techniques. With this knowledge, preventive actions can be put in place that are specific to each person's genetic makeup and way of life [70]. It is possible to reduce risk factors and postpone the beginning of disease by recommending dietary changes, cognitive exercises, and lifestyle modifications. By choosing volunteers for clinical trials according to genetic or molecular factors, precision medicine helps to improve trial efficacy. This method increases the possibility of determining which people will gain the most from a given intervention. By discovering focused therapeutic methods, precision medicine expedites the discovery of new drugs and may result in more effective and successful trials [78].

### 3.2. Targeted Therapies and Personalized Interventions Based on Genomic Profiles

Undoubtedly, numerous neurodegenerative disorders, exhibit clinical heterogeneity due to a substantial genetic influence. Consequently, these diseases manifest diverse clinical courses, thereby precluding the universal application of identical treatments to patients afflicted with identical pathology [79]. Precisely due to the diverse molecular characteristics exhibited by these subgroups, the current objective of scientists and physicians is to identify and catalog every single variant. The overarching goal is subsequently to comprehend the molecular underpinnings of neurodegenerative disorders and develop efficacious targeted treatments [76]. The emergence of personalized medicines and customized interventions that capitalize on the distinct features of individual patients' genetic composition can be attributed to this paradigm shift. The field of disease management has changed because of the application of genomics in clinical practise, which offers more potent medications with fewer adverse effects [68]. Cancer treatment is one of the main areas where specific medicines based on genetic profiles have shown impressive results. Conventional cancer treatments frequently affect both malignant and healthy cells systemically. Through the analysis of genetic variations particular to a patient's tumor, physicians can pinpoint specific weaknesses that can be precisely targeted [80]. Tyrosine kinase inhibitors and immunotherapies, which specifically target cancer cells while minimizing damage to healthy tissues, are two examples of medicines that have been developed because of this. Finding useful mutations in cancer cells has become essential to oncology decision-making when it comes to treatment. For instance, the usage of PARP inhibitors in the treatment of breast and ovarian cancers may be influenced by the existence of specific genetic abnormalities, such as those found in the BRCA genes. Like this, the identification of gene fusions or mutations can direct the administration of targeted treatments in lung cancer, such as EGFR inhibitors [76]. Targeted medicines based on genetic profiles are emerging in other therapeutic areas, not just cancer. Genetic testing can be used to identify people who have inherited issues that make them more susceptible to cardiac problems in the context of cardiovascular diseases. Then, to better control cardiovascular risks, tailored therapies such as lifestyle changes and prescription drugs can be used [71].

Many neurological disorders have no approved treatment, but tailored therapies can reduce the course of the illness and enhance the quality of life for those who are affected [68]. When it comes to infectious diseases, genomics is essential to the creation of focused antiviral drugs. Researchers can find unique vulnerabilities that can be used for therapeutic purposes by examining the genetic makeup of diseases. This strategy has proven especially useful in the development of antiretroviral medications for HIV, as knowledge of the virus's genetic diversity has allowed for the formulation of extremely successful and individualized treatment plans [77]. There are still difficulties even with the proven benefits of tailored interventions and focused therapies. Some of the challenges that require cautious navigation are the availability of genetic testing, ethical considerations, and the interpretation of complex genomic data [78]. To actualize the potential of precision medicine in neurological disease, however, numerous specialized centers must engage in collaborative efforts across diverse domains of fundamental and translational research to execute precision medicine strategies. The establishment of these networks will enable the dissemination of information derived from various studies and facilitate the accumulation of data. Many samples from healthy individuals, who can provide control data for comparison with those of patients, are also crucial in this context. Through the integration of genetic data, clinic records, and patient images, a progressive resolution of the enigma will be achievable, thereby facilitating a more comprehensive understanding of the ailment. Concerning this matter, the "Italian IRCSS Network of Neuroscience and Neurorehabilitation" serves as an exemplary web-based platform. Its primary objective is to optimize and standardize the therapeutic approaches utilized for neurodegenerative diseases and the clinical care provided to patients [81].

**Table 2:** Description of few environmental factors having great impact on Dementia.

Environmental Factor	Influence on Dementia	Reference
Education	Higher levels of education are associated with a reduced risk of dementia.	[84]
Physical Activity	Regular physical exercise is linked to a lower risk of cognitive decline and dementia.	[85]
Social Engagement	Maintaining social connections and engagement may contribute to cognitive health and reduce dementia risk.	[86]
Diet	A healthy diet, such as the Mediterranean or DASH diet, is associated with a lower risk of dementia.	[87]
Vascular Health	Conditions affecting vascular health, like hypertension and diabetes, can increase the risk of VaD.	[88]
Traumatic Brain Injury (TBI)	Severe head injuries, especially repeated concussions, have been linked to an increased risk of dementia later in life.	[89]
Air Pollution	Long-term exposure to air pollution has been suggested as a potential risk factor for cognitive decline and dementia.	[90]
Smoking	Smoking is associated with an increased risk of dementia, possibly due to vascular damage and other harmful effects on the brain.	
Alcohol Consumption	Excessive alcohol consumption is a risk factor, while moderate alcohol intake may have some protective effects on cognitive function.	[91]
Sleep Quality	Poor sleep patterns and disorders have been linked to an increased risk of cognitive decline and dementia.	[92]
Depression and Stress	Chronic stress and depressive symptoms may contribute to cognitive decline and increase the risk of dementia.	

## 4. Gene-Environment Interactions in Dementia

### 4.1. Environmental Factors Influencing Dementia

Recent studies point to a connection between prolonged exposure to air pollution and a higher chance of dementia and cognitive decline. Fine particulate matter and nitrogen dioxide are two examples of airborne pollutants that can enter the bloodstream and travel to the brain, where they may cause oxidative stress and inflammation. Long-term exposure to contaminated air may accelerate the onset of neurodegenerative diseases [79]. Underneath Table 2. Speaks about a few environmental factors that directly or indirectly influence Dementia. The risk of dementia has been linked to occupational exposure to some chemicals, including pesticides and heavy metals. Regular exposure to neurotoxic compounds is a need for several vocations, and over time, this accumulation of toxins in the brain can impair cognitive function. The study by Lourida et al. [82] reveals the following as key findings: (I) irrespective of genetic risk, an unfit lifestyle increases the risk of developing dementia; and (II) a healthy lifestyle can reduce the risk of developing dementia, independent of genetic risk. It is essential to note, however, that while adopting a healthy lifestyle may reduce the risk of incident dementia for those with a high genetic risk score, it does not eliminate the risk of developing dementia. The authors observe that among individuals who have a high genetic risk of developing dementia, the distinction between maintaining a healthy or unhealthy lifestyle prevents the disease in approximately one in every 121 individuals over ten years. The specific impact of lifestyle behaviors on the severity or time-to-onset of dementia was not investigated. Considering the finding in this study that a healthy lifestyle reduced, but did not eliminate, the genetic contribution to dementia risk, it is worthwhile to investigate whether therapeutics can enhance the effects of a positive lifestyle in genetically susceptible individuals [82]. The health of the brain is greatly influenced by diet, and there is a substantial correlation between nutritional factors and dementia risk. Antioxidant, omega-3 fatty acid, and vitamin-rich diets have been associated with a decreased risk of cognitive deterioration [80]. When conducting ketogenic investigations in rodents, exogenous ketones are commonly used. Numerous studies have shown that AD-transgenic mice that were administered ketones exhibited enhanced cognitive function and a reduced β-amyloid burden [83]. In a similar vein, a minor clinical trial involving human subjects diagnosed with moderate cognitive impairment (MCI) demonstrated that verbal memory could be enhanced through the implementation of a low-carbohydrate (ketogenic) diet. Despite the encouraging nature of these findings, there are significant obstacles that must be surmounted to utilize the therapeutic benefits of ketones in the treatment of dementia [84]. A diet heavy in processed foods and saturated fats may raise the risk of dementia by causing vascular issues and inflammation [85], as indicated by a large population study conducted in Norway, those who abstained entirely or consumed alcohol more frequently had higher HR ratios (1.30 and 1.45, respectively) for dementia and AD compared to those who drank infrequently. Analogous findings were presented in an additional cohort, wherein heavier consumers who frequently consumed hard liquor experienced a more pronounced decline in cognitive function compared to those who consumed wine or alcohol less frequently [86]. Dementia risk has continuously been linked to regular physical activity. Exercise improves neuroplasticity, lowers inflammation, and strengthens the heart. Conversely, sedentary lifestyles are associated with a higher risk of cognitive deterioration. Throughout life, consistent physical activity can help to maintain general brain resilience [81].

Dementia risk factors include social isolation and low levels of cognitive stimulation. Keeping up social relationships, partaking in mentally challenging pursuits, and continuing education are all ways to support cognitive reserve, which helps the brain adapt to aging and lowers the risk of dementia [87]. Although dementia is not caused by aging per se, the likelihood of developing the condition increases as one ages. The correlation between aging and dementia is intricate, with numerous factors playing a role in this relationship such as Neurodegenerative changes, Environmental factors, genetic factors, vascular changes, and accumulation of pathological protein [88]. The risk of cognitive impairment has been linked to poor sleep quality and illnesses such as sleep apnea [89]. For the brain to function properly and to consolidate memories, sleep is essential. Prolonged sleep disruptions may be a factor in the build-up of beta-amyloid plaques in the brain, which is a characteristic feature of AD. Dementia risk has been related to cardiovascular variables, such as high cholesterol, diabetes, and hypertension. Heart-healthy practices frequently benefit the brain as well. VaD and cognitive decline can be exacerbated by conditions that impair blood flow and vascular health. A substantial risk factor for dementia is a history of traumatic brain damage, particularly recurrent head trauma, especially in disorders such as chronic traumatic encephalopathy (CTE) [90].

### 4.2. Dynamic Interplay between Genes and the Environment

Gene-environment interactions are categorized according to the way by which distinct genotypes influence the disease risk induced by environmental exposures or lifestyle factors. These phenomena are highly significant, yet inadequately investigated, in a disease as intricate as AD, given that the condition manifests in numerous complex forms and potentially has a multiplicity of aetiologies [91]. The acknowledgment of the multiplicity of aetiologies of AD represents a key challenge in moving toward the goal of targeted interventions or treatments for this complex multifactorial disorder. AD is characterized by intricate and heterogeneous pathogenic processes that involve genetic, environmental, and lifestyle components, but also by complex interactions among these components thus warranting the need for understanding a spectrum of mechanisms underlying pathogenesis in the development of efficacious interventions. One of the primary challenges posed following the acknowledgment of the multiplicity of aetiologies of AD is the heterogeneity seen among affected individuals. AD is manifested by various clinical phenotypes that range from early-onset familial forms with clear genetic determinants to late-onset sporadic forms with multifactorial origins. This heterogeneity complicates the development of targeted treatments that cater to the specific needs of the subsets of affected individuals. Furthermore, the multitude of comorbidities and the overlapping pathophysiological cascades in AD further confound the task of identifying highly specific therapeutic targets. Another major implication of the multiplicity of aetiologies of AD is the lack of a single disease model that encompasses all facets of the pathogenesis of AD [92]. AD is associated with the accumulation of amyloid-beta plaques, tau tangles, neuroinflammation, synaptic dysfunction, and neuronal loss among other pathologies and these various mechanisms likely potentiate the progression of the disease in differing degrees in different individuals thus raising the need for personalized treatment approaches guided by an individualized profile of the disease. However, promising therapeutic avenues in targeting neuroinflammation, synaptic dysfunction, or mitochondrial dysfunction in AD are emerging from recent efforts in defining specific subgroups of affected individuals that may be more appropriately targeted for these specific interventions based on their unique genetic and environmental profile in precision medicine or a personalized treatment approach [2].

Identifying singular genetic risks in complex diseases like late-onset AD is uncommon, and most genetic findings, except for APOE, manifest as modest responses. These observations suggest that additional risk factors are more likely to interact with or be associated with this genotype [92]. Individual characteristics, behaviors, and sensitivity to different conditions are shaped by this complex interaction. Comprehending the interplay between genes and the environment is crucial in deciphering the intricacies of human biology, impacting domains ranging from genetics and psychology to medicine and public health. Parents pass on their genes, which are the sections of DNA that contain instructions on how to construct and maintain the body [93]. They are fundamental in forming an individual's features, ranging from eye color to illness susceptibility. The genetic differences among individuals are attributed to variations in DNA or polymorphisms. Although genes offer a blueprint, environmental circumstances have a significant impact on how genes express themselves. The study of changes to gene activity that do not involve changes to the underlying DNA sequence is known as epigenetics. Without changing the genetic coding, environmental influences can create epigenetic alterations that affect gene expression. Epigenetic processes, such as DNA methylation and histone modification, regulate gene function and are susceptible to environmental influences. An organism's growth trajectory is determined by the interaction between genes and environmental stimuli during important developmental stages [67]. The ability of an organism to modify its structure and function in response to external stimuli is known as developmental plasticity. This phenomenon is seen at many phases of life, including the beginning of life and embryonic development, when environmental factors can have a significant and long-lasting impact on health and wellbeing [94]. There are gene-environment interactions associated with numerous lifestyle factors in AD and dementia. A longitudinal study was conducted to investigate the relationship between physical activity and APOE4 carriers in elderly patients (75 years of age). Participants were evaluated for dementia and re-evaluated 4.5 years later. Higher levels of physical activity were associated with a reduced risk of dementia and AD. A combination of a low level of physical activity and an APOEε4 allele significantly increased the risk of dementia in individuals compared to the effects of either factor alone [92]. Longitudinal epidemiological research, the Honolulu-Asia aging study examined the relationship between risk factors and dementia and cognitive decline in Japanese American men. The authors present their findings that smoking was associated with a 2.18-fold increased risk of dementia in comparison to nonsmokers; this association was not influenced by APOE genotype. The rs7179008 variant of CHRNA7 (α7 nicotinic acetylcholine receptor) was found to reduce the risk of LOAD (AOR=0.29) and avert the heightened risk associated with smoking in LOAD, according to another study [92].

Gene-environment interactions have a major impact on health and illness susceptibility. While certain people may be predisposed to specific disorders due to genetic variances, environmental variables frequently influence how these conditions appear. For instance, a person who is genetically predisposed to diabetes may not get the disease until they are exposed to lifestyle variables, such as eating poorly and being sedentary all the time [95]. Both the environment and genes play a role in the development of behavioral traits and personality traits. Environmental factors, such as upbringing and cultural influences, impact behavioral tendencies, whereas genetics may influence qualities like temperament and impulsivity. Complex qualities like intellect, where both genetic predisposition and environmental variables influence, are good examples of the interplay between nature and nurture [95]. To investigate and comprehend how the environment can potentially interact with the genetic profile of an individual, it is essential to possess a fundamental comprehension of the environmental hazards in question, in addition to the prevailing genetic risk factors. The importance of considering additional risk factors, such as age, sex, and familial history of dementia, when analyzing an individual's unique risk signature has become even more apparent [95].

## 5. The Nexus of Genomic Insights, Precision Medicine, and Gene-Environment Interactions

Our understanding of complex diseases is changing dramatically because of the convergence of genomic findings, precision medicine, and gene-environment interactions. This is especially true when it comes to neurodegenerative disorders like dementia [1]. Numerous genetic variants have been linked by genomic studies to an increased risk of dementia, laying the groundwork for precision medicine strategies that customize treatments to each patient's unique genetic profile [1]. Particularly, the APOE gene has been thoroughly investigated concerning AD, and the APOEε4 allele is a significant genetic risk factor. Other genes associated with family variants of AD include PSEN1 and PSEN2 [88]. By acknowledging the diversity of genetic factors that contribute to dementia, precision medicine makes use of these genomic discoveries to optimize therapeutic approaches. Specifically, pharmacogenomic considerations are essential for customizing medication regimens according to a patient's genetic composition [96]. For example, changes in the CYP2D6 gene can affect the cholinesterase inhibitors, which are frequently used to treat AD symptoms, are metabolized. Comprehending the subtleties of pharmacogenomic medicine enables tailored drug choices and dosages, enhancing therapeutic effectiveness while reducing adverse effects. The precision medicine strategy is extremely complicated by the complex interactions between genes and the environment. The onset and course of dementia are markedly influenced by gene-environment interactions. Genetic predispositions combine with environmental factors, such as diet, lifestyle, and exposure to pollutants, to shape an individual's risk profile [64]. For example, a study by Aravena et al. [109] showed that exposure to high levels of air pollution may exacerbate cognitive impairment in people who carry the APOE ε4 gene, which is genetically predisposed to AD. This emphasizes how crucial it is to take the environment into account when creating precision medicine plans for dementia [109]. The development of cutting-edge technologies like single-cell sequencing and next-generation sequencing (NGS) has greatly expanded our knowledge of molecular interactions between genes and environments. With the use of NGS, whole genomes may be thoroughly analyzed, which makes it possible to find uncommon variants and conduct a more in-depth investigation of the genetic terrain connected to dementia [110]. Contrarily, single-cell sequencing offers information about cellular heterogeneity and makes it possible to identify cell types that might be more vulnerable to outside factors [111]. By enabling researchers to disentangle the complex relationships between genetic and environmental factors, these tools advance our understanding of the genesis of dementia from a more comprehensive perspective [112]. Establishing people's understanding of the consequences of genomic testing and how it might affect their healthcare choices is crucial to fostering confidence in precision medicine practices [113]. This strategy is fostered and improved by the complex interconnections between genes and the environment, which acknowledge that the dynamic interactions between genetic predispositions and environmental circumstances determine how effective interventions are. The incorporation of gene-environment interactions into precision medicine shows promise for more efficient, individualized approaches to dementia prevention and treatment as technology develops and our understanding expands [99].

**Figure 2.**
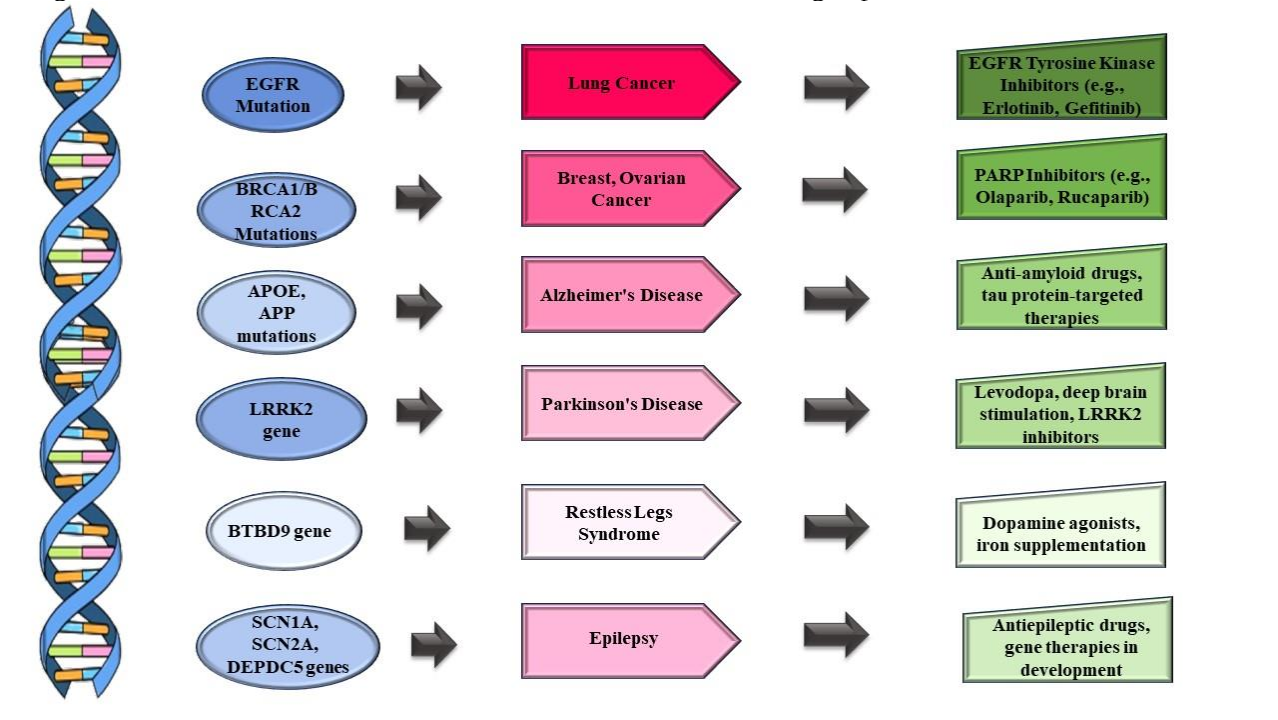
Schematic demonstration of few genes (Blue) involved in specific diseases (Pink) with their targeted therapies (Green).

## 6. Translational Implications

### 6.1. Applying Genomic Insights to Develop Targeted Therapies

A significant development in the medical field is the use of genetic knowledge to generate targeted medicines. Treatment can be customized according to genomic information, which offers a thorough insight into a person's genetic composition. Figure 2. speaks about several genes of diseases and their targeted therapies. Precision medicine, which offers more individualized and effective therapeutic approaches, has the potential to completely change the healthcare industry by replacing the one-size-fits-all paradigm [99]. The detection of mutations, variations, and other genetic anomalies that contribute to the onset and progression of a variety of ailments, such as cancer, genetic disorders, and complicated diseases, has been made possible by advancements in genomic sequencing technologies [113]. Genomic insights have completely changed our understanding of cancer biology in oncology. More accurate classification of cancer subtypes has been made possible by the discovery of oncogenes, tumor suppressor genes, and driving mutations. With the help of this knowledge, targeted cancer medicines can be developed by customizing treatment based on the distinct genetic profile of an individual’s tumor. In the case of cancer, targeted therapies particularly attempt to hinder the molecular mechanisms that propel cancer cell division and survival [113]. Through the identification of genetic changes that render cancer cells susceptible to medications, genomic insights inform the selection of targeted therapies. For instance, trastuzumab and other HER2-targeted treatments work well for breast tumors that have an amplified HER2 gene [108].

To find inherited genetic alterations linked to illnesses like hereditary malignancies, neurological problems, and cardiovascular ailments, genomic insights are used in genetic testing and diagnostics. Risk assessment, tailored preventative tactics, and proactive interventions are made possible by the early identification of genetic predispositions. This field of study looks at how a person's genetic makeup affects how they react to drugs [106]. Based on a person's genetic profile, genomic insights inform medicine selection and dosage, maximizing therapeutic benefits and reducing side effects. This strategy is especially important in medical specialties like psychiatry, where differences in medication metabolism might affect the effectiveness of treatment. Understanding uncommon genetic illnesses is greatly enhanced by genomic insights [70]. The development of tailored medicines for uncommon diseases is made possible by the discovery of the underlying genetic abnormalities. Sometimes, the exact genetic anomalies causing the disease can be addressed by orphan medications, which are meant to treat certain disorders. A novel method of treating cancer, immunotherapy uses the body's immune system to find and destroy cancer cells. The identification of genetic markers that are predictive of a tumor’s sensitivity to immunotherapies is made possible using genomic insights. Certain tumors have responded remarkably well to this individualized approach of treatment [94].

### 6.2. Personalized Treatment Plans Based on Genomic and Environmental Profiling

In the context of dementia, personalized therapy regimens based on environmental and genetic analysis constitute a cutting edge in neuroscientific research. The combination of environmental influences, genetic complexity, and genomic data opens new possibilities for customizing interventions that consider the complexities of each patient's disease [105]. Since different types of dementia have been linked to distinct genetic variables, the field of genomic profiling in dementia has received significant traction. The most prevalent type of dementia, AD, has been intensively examined for the APOE gene. One important genetic risk factor for AD is the APOE ε4 allele, which is carried by one or two copies of the allele, increasing the likelihood of the illness developing and frequently causing symptoms to appear earlier. By identifying those who are more likely to experience problems, early interventions, and more focused therapeutic approaches can be implemented by incorporating this genetic data into individualized treatment programs [88]. A more comprehensive understanding of the underlying mechanisms of dementia has been made possible by the discovery of other genetic variables linked to the disease, beyond APOE. For example, the pathophysiology of AD and some forms of FTD involves mutations in genes linked to the processing of APP and the formation of tau protein tangles, such as those associated with the PSEN and MAPT genes, respectively. By incorporating genetic variation information into individualized therapy strategies, it is possible to target biological pathways and address the underlying causes of the disease [99, 96].

Our ability to identify and understand additional genetic risks beyond the APOE gene has significant implications and challenges for research and clinical practice in AD. The APOE gene remains the best-established genetic risk factor for late-onset AD but recent advances in genomic research have identified numerous additional genetic variants that modestly contribute to the risk of developing this common and devastating brain disease. Deciphering the functional significance of these variants and their relevance to the pathogenesis and treatment of disease raises several daunting challenges. That the genetics of AD is complex and heterogeneous is one of these [114]. GWAS in recent years have uncovered well over 30 genetic loci associated with late-onset AD. Each contributes modestly to disease risk, and most are clustered tightly near one other in the genome. Moreover, these are in genes implicated in diverse processes including amyloid-beta metabolism, tau phosphorylation, neuroinflammation, synaptic function, and cholesterol metabolism. How these genetic variants impact these and other biological processes, how they interact with one another, and how they interface with environmental factors that influence the risk of disease, is not easily or immediately understood. The causal relevance of many of these additional genetic risk factors to the pathogenesis of disease is not immediately clear. While GWAS have been highly effective in identifying susceptibility loci, the functional relevance of most associated genetic variants is not known. Distinguishing those variants that directly contribute to the pathogenesis of AD from those that merely mark nearby functional variants using sophisticated experimental and computational approaches is a central challenge [18]. Furthermore, the significant genetic heterogeneity across human populations further complicates the interpretation of genetic data. The additional identification of genetic risks beyond the APOE gene highlights the need for an individualized treatment approach based on everyone’s unique genetic profile. While APOE genotyping can inform risk assessment and prognosis in the clinic, the integration of additional genetic risk factors into these risk prediction models could make them even more accurate. But translating genetic information into actionable clinical insights is challenging, particularly given the modest effect sizes of most genetic variants, and the highly complex interplay between genetic and environmental factors in the pathogenesis of AD [115].

Environmental variables can have a significant impact on how dementia manifests and advances. Lifestyle factors that have been linked to impacting the risk and course of the disease include food, physical activity, and cognitive engagement. Exercise has been demonstrated to have neuroprotective effects, and a diet high in antioxidants and omega-3 fatty acids has been linked to a lower incidence of dementia [114]. Exposure to some environmental factors, like air pollution and pollutants, has also been associated with a higher chance of cognitive deterioration. Understanding the unique risk profile for dementia is made more complete when environmental factors are considered in addition to genetic data [116]. It has the potential to customize interventions to target the cellular and molecular processes connected to a person's cognitive decline [117]. New developments in neuroimaging methods, such as positron emission tomography (PET) and functional magnetic resonance imaging (fMRI), make it possible to evaluate the structure and function of the brain, leading to a more accurate diagnosis of the disease at the individual level. For instance, choosing targeted medicines targeted at modifying disease processes can be aided by determining the unique patterns of brain atrophy or aberrant protein accumulation in a dementia patient [117].

While creating individualized treatment regimens for dementia, pharmacogenomic factors are essential. Appropriate treatment regimen selection can be guided by knowledge of the process of genetic variants affecting pharmaceutical response [108]. This is especially important when it comes to symptomatic therapies for cognitive decline since different people react differently to drugs like cholinesterase inhibitors and N-methyl-D-aspartate (NMDA) receptor antagonists [96]. Clinicians can enhance drug selection and dosages through personalized treatment regimens that are based on pharmacogenomic findings. This approach can maximize the effectiveness of pharmacological interventions while avoiding potential negative effects [1]. Certain lifestyle changes, like cognitive training, exercise, and dietary adjustments, have demonstrated potential in mitigating cognitive decline and enhancing cognitive performance in certain dementia patients. Adapting these approaches to a person's unique genetic and environmental background may increase their efficacy even more. It highlights the significance of customized lifestyle recommendations because those with a genetic predisposition to inflammation, for example, may benefit more from anti-inflammatory dietary therapies [118]. Healthcare experts such as neurologists, geneticists, and neuropsychologists work together to evaluate and integrate complicated genomic and environmental data. To find significant correlations and create predictive models for each patient's unique treatment response, advanced data analytics and artificial intelligence (AI) are essential tools for analyzing patterns within large datasets [83].

Personalized dementia treatment approaches require careful consideration of ethical issues. Consent, privacy, and the possibility of stigmatization due to genetic information are all issues that need to be carefully considered. Building trust in the scientific and medical communities requires making sure people are aware of the implications of genomic testing and how their data will be utilized [119]. To protect the rights and welfare of people taking part in individualized treatment research, legal and ethical standards need to keep up with technical development. Individualized therapy regimens based on genetic and environmental profiles hold the potential to be more focused and successful interventions, potentially delaying the onset of dementia and enhancing the quality of life for those who are impacted [120].

### 6.3. Challenges and Opportunities in Translating Genomic Findings to Clinical Practice

There are many potentials as well as daunting hurdles in the complicated terrain of translating genetic results into clinical practice for dementia. Dementia is inherently heterogeneous, with a multitude of genetic and environmental factors contributing to both its development and progression. This presents one of the main obstacles. Numerous susceptibility genes associated with distinct types of dementia have been found through genomic research; nevertheless, comprehending how these genetic factors interact and impact the disease's varied expressions is still a major challenge [121]. Many genetic variations linked to dementia still lack a clear understanding of their functional implications, which makes it difficult to design targeted medicines that address the underlying causes of the illnesses [122].

Logistical and ethical issues arise when integrating genetic data into standard healthcare practice. Complicated decision-making procedures, such as gaining informed consent and addressing privacy concerns, are involved in genetic testing for dementia risk. The ethical use of genetic data to avoid prejudice and the possible psychological effects on individuals and their families calls for careful consideration during the translation process [107]. It is critical to safeguard patients' genetic information against unauthorized access and misuse, as it is highly sensitive. Patients may encounter difficulties in providing informed assent due to their limited understanding of the intricacies of genomic data and its ramifications. It is critical to ensure equitable access to precision medicine to prevent the worsening of pre-existing healthcare disparities [97]. Ensuring patient rights are protected while scientific knowledge is advanced is a critical consideration for the ethical implementation of precision medicine in dementia research and care [97,98]. To improve the consistency and reproducibility of results and facilitate their therapeutic utility, standardized methods, and recommendations for genomic testing in dementia must be developed and put into practice [95]. Though, these obstacles, applying genetic research to clinical practice presents several benefits, especially in the areas of early diagnosis and customized care. The foundation for the creation of prediction models that may allow for the early identification of those at higher risk before the onset of symptoms has been laid by the discovery of genetic markers linked to dementia risk [83]. Successful translation requires cooperation between genetic counselors, physicians, and researchers. A crucial role is played by interdisciplinary teams that are adept at navigating the intricacies of genetic data and effectively communicating findings to patients and healthcare professionals [123,124].

## 7. Emerging Technologies Shaping the Future of Genomic Research

To accelerate the application of the precision medicine paradigm to a broader spectrum of complex diseases, numerous governments around the world support the implementation of Precision Medicine Initiatives (PMIs). These endeavors are of great importance as they aim to generate the extensive corpus of scientific knowledge necessary for groundbreaking developments in early detection, prevention, and treatment, in addition to efficiently integrating the notion of precision medicine into regular clinical practice [125]. On 20 January 2015, U.S. President Barack Obama unveiled the Precision Medicine Programme Cohort Programme, a research endeavor aimed at accelerating the adoption of a novel generation of precision medicine (PMI-CP). Details regarding this program are available at Precision Medicine Initiative | The White House (www.archives.gov/, accessed on 1 July 2022). It is anticipated that over a million Americans will participate in the research cohort [1]. This demographic will be requested to provide informed consent for the comprehensive characterization of biological specimens and behaviors, all of which are associated with electronic health records (EHRs). Systematically amassing extensive, intricate, and profound data [125] will enable the implementation of observational studies pertaining to technologies and medications and may even facilitate more rigorous interventional research targeting specific issues. Despite an initial focus on significant disease areas, such as cancer, this strategy is designed specifically to target brain diseases [125].

**Figure 3.**
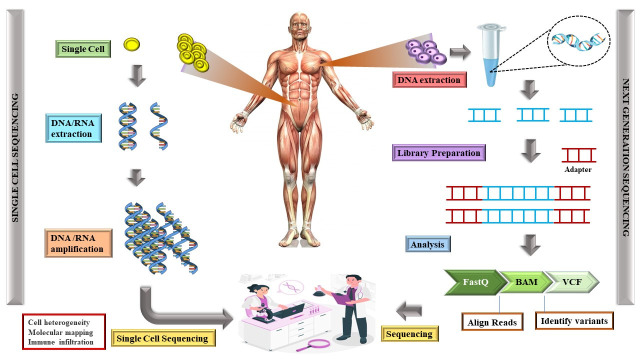
Schematic illustration of Single Cell Sequencing concept and Next Generation Sequencing.

A new age in dementia genomic research is being ushered in by emerging technologies, which provide unparalleled insights into the intricate interactions between neurodegenerative illnesses and genetics. Next-generation sequencing (NGS) is a major technique that is advancing things, as shown in Figure 3. Due to the rapid and affordable genome sequencing made possible by NGS technology, researchers can now thoroughly investigate the genetic landscape linked to dementia. With the help of NGS's high-throughput capabilities, uncommon genetic variants, common polymorphisms, and structural differences can be identified, leading to a more thorough understanding of the genetic variables influencing dementia susceptibility [101]. Another innovative technique that is revolutionizing the field of dementia genomics research is single-cell sequencing. Conventional genomic techniques frequently analyze tissue in bulk, which hides cellular heterogeneity [100]. However, single-cell sequencing gives scientists the ability to examine genetic differences at the individual cell level, providing a more nuanced picture of the various cell populations found in the brain (Figure 3). Studying complex tissues like the brain, where cellular diversity is essential to neurodegenerative processes, is where this approach is very useful [97]. The complexities of genetic contributions to dementia are being unraveled at a resolution never before possible because of single-cell genomic studies, which shed light on cell-specific gene expression, mutations, and epigenetic alterations [103]. The future of dementia genomics research is also being shaped by developments in imaging technologies. Through the integration of genomics with neuroimaging modalities like PET and fMRI, researchers can connect genetic differences to patterns of brain activity and structural alterations [104]. This multimodal method offers a comprehensive understanding of how hereditary variables affect the morphological and functional features of the brain in dementia. To better understand the genetic basis of dementia symptoms, researchers can investigate, for example, how particular genetic variations are linked to the formation of aberrant protein aggregates or changes in neural connections [100].

In the field of dementia genomic research, AI, and machine learning (ML) are quickly becoming essential instruments. Large datasets can be analyzed by these technologies, which can also spot patterns and forecast relationships that could be missed by more conventional analytical methods [126]. AI and ML are used in genomics to decipher complex genomic data, find new genetic markers, and forecast illness risk based on a person's genetic profile. The integration of various datasets, including genomes, clinical records, and imaging data, is being facilitated by AI-driven algorithms, offering a more thorough and integrated method of comprehending the genetic architecture of dementia [105]. Laboratory manipulation of specific genes has been revolutionized by CRISPR-Cas9 gene editing technology. Researchers studying dementia may now precisely, alter genes linked to neurodegenerative diseases via CRISPR-Cas9, which offers important new information about the functional effects of genetic variants. Due to this technology, it is possible to create cellular and animal models of dementia that more closely mimic its hereditary components. This makes it easier to research the disease's causes and create tailored treatment therapies [106]. Even with these advancements in technology, problems still exist. In the era of advanced genomics, ethical concerns—like protecting data privacy and consent—are crucial. As these technologies develop, the combination of advanced methodologies and genomics holds great promise for understanding the complexities of dementia and, eventually, opening the door to more focused diagnostics and creative treatment approaches [107]. Ensuring the trust and well-being of participants in individualized treatment research for dementia hinges on addressing ethical concerns related to consent, privacy, and potential stigmatization of individuals. The resolution of these concerns will require a comprehensive approach that is grounded in transparency, respect for autonomy, robust data protections, and stigma reduction. Below, we offer several strategies for tackling these ethical challenges [127,128].

### 7.1. Informed Consent

Prioritizing informed consent is critical to ensuring that individuals understand the nature of their participation in individualized treatment research for dementia. This entails providing detailed information about the study’s purposes, anticipated risks and benefits, data collection procedures, and participants’ rights. Informed consent should be obtained without coercion, and individuals should have opportunities to ask questions and to leave the study at any time [129].

### 7.2. Privacy Protection

A key concern for individualized treatment research for dementia is protecting the privacy and confidentiality of participants’ personal and health information. This will involve the implementation of rigorous data security measures — such as encryption, access controls, and anonymization- to prevent unauthorized access to and the disclosure of sensitive information. Researchers will also need to follow any applicable data protection regulations, including, for example, the General Data Protection Regulation (GDPR), to ensure the ethical and lawful processing of personal data [113].

### 7.3. Minimizing Stigmatization

Individualized dementia treatment approaches may involve genetic testing or the collection of sensitive health information, which could lead to stigmatization or discrimination. Efforts to destigmatize dementia and raise awareness and understanding about the condition may mitigate this risk. Healthcare providers and researchers should also strive to foster non-judgmental attitudes and to connect individuals with dementia and their families to supportive services in the community. In addition, policies and laws that prevent discrimination based on genetic predisposition or health status should prevent this additional stigmatization of participants in individualized treatment research for dementia [130].

### 7.4. Empower Participants

Providing individuals with the information and support needed to allow them to make informed choices about participation in individualized treatment research is essential. This will likely involve education and programs to support self-advocacy and knowledge of participant rights and preferences. Including individuals in research in various capacities, such as participating in patient advisory groups or through community-based participatory research strategies, will help assure that the voices and perspectives of individuals and caregivers with dementia are heard and honored [131].

### 7.5. Ethical Oversight

The establishment of rigorous ethical oversight to protect the rights and welfare of dementia treatment research participants also will be essential. Institutional review boards or research ethics committees should carefully review study protocols, consent procedures, and data management approaches considering the unique contextual and participant-related circumstances that can arise in individualized treatment research for dementia [132].

Ongoing research into targeted therapies and novel biomarkers holds great promise for the future of precision medicine in AD. Potentially enhancing the precision of diagnoses, the application of precision medicine could result in earlier intervention and, consequently, improved outcomes [1]. The classification of AD subtypes using genetic biomarkers is a promising area of study because it would enable the development of more targeted treatment strategies. PET scans and other novel imaging modalities are currently being employed to detect biomarkers indicative of the existence of amyloid plaques and tau tangles within the brain [126]. Additionally, precision medicine may facilitate the creation of novel treatments for dementia. Through the process of subtyping the disease, scientists can concentrate their efforts on the development of therapies that specifically target the root causes of that subtype. This could potentially result in the creation of more efficacious treatments that include fewer adverse effects [1]. The application of ML techniques to precision medicine is a significant development. This may contribute to a more precise diagnosis and early detection of dementia. In brief, precision medicine possesses the capacity to fundamentally transform the pathogenesis and management of dementia, resulting in enhanced therapeutic interventions and improved patient prognoses [133].

## 8. Conclusion

The incorporation of precision medicine into contemporary healthcare policies and practices pertaining to dementia signifies a paradigm shift in the methods of diagnosis and treatment. Healthcare systems must invest in sophisticated genomic technologies and establish protocols for accumulating, analyzing, and interpreting genetic data to achieve seamless integration. It is crucial to provide healthcare professionals with training on how to comprehend and effectively employ precision medicine tools so that they may incorporate genomic insights into the provision of dementia care [1]. It is imperative to establish standardized guidelines and protocols to promote uniformity throughout healthcare environments and streamline the integration of precision medicine into customary diagnostic and therapeutic approaches. Undoubtedly, the integration of technological and theoretical advancements—such as System biology (SB), genomic sequencing, exploratory high throughput evaluations, the introduction of biological markers, records and computational methods, interconnected disease modeling, EHRs, intelligent systems, —has established precision medicine as one of, if not the primary, innovative objectives. In this context, the PMI-CP was declared official by the U.S. President as stated above [1].

To sum up, the analysis of genetic insights into dementia is a significant step in the right direction toward understanding the complexities of this crippling illness. With precision medicine, personalized treatments based on each person's own genetic profile become possible, offering a glimmer of hope. The interaction between genes and the environment adds a layer of complexity that necessitates more research. The consequence of the gene-environment interaction highlights the obligation of a comprehensive strategy for comprehending and treating dementia. The application of genomic data to clinical practice has the potential to revolutionize care in the future. Tailored treatment regimens based on a thorough understanding of a person's genetic predispositions and environmental exposures may mark the beginning of a new era in dementia care. However, caution should be taken while navigating ethical issues, data protection, and the difficulties of applying such complex information widely. To translate genomic insights into real changes in the lives of people living with dementia and their families, academics, healthcare providers, and politicians must work together relentlessly in the pursuit of effective solutions.

## Data Availability

No new data were created or analyzed in this study. Data sharing is not applicable to this article.
